# The Colonial Lunatic Asylum, Berbice

**Published:** 1897-12-25

**Authors:** 


### THE COLONIAL LUNATIC ASYLUM, BERBICE.

This is situated a pleasant walk from New Amsterdam, and is the only establishment for the insane in the colony; it stands on the point near the junction of the Canje and Berbice rivers. The grounds are extensive and well-drained, and include large provision gardens, where plantain, cassava, sweet potatoes, and other things are grown. The principal entrance is by a pretty little gatehouse, but there is access to doctors' houses from the grounds. The buildings themselves are mostly old, being part of the old barracks built by the Dutch, and as far as possible accommodated to their present use, the old guard house being used as a bakery.

Criminal and pauper lunatics are all sent here, as well as any other cases. The average number of inmates is between 600 and 700. The majority are coloured and black, with a large sprinkling of coolies, and Portuguese and a few Europeans. The nurses and various attendants are well disciplined and, generally speaking, are neat and clean in appearance. Female attendants wear holland or white uniforms, with bands of blue on the skirt, the number of bands indicating years of service. Merit and good conduct badges are worn on the sleeve. Generally speaking, the male attendants are of a superior class to the female and, I hear, more reliable. Most of the nurses and attendants live outside. Day warders are on duty from six a.m. to six p.m.; night six p.m. to six a.m. Their work is hard in some cases, and without strict regulations neglect would soon slip in; breaches of rules are punished by fines. The male inmates have a fine large day and dining room, which is used for fortnightly dances, to which the public are sometimes admitted.

It is a pleasant sight to see the men at dinner—tables spotlessly clean, spoons bright, bowls and plates pure white. The soup is brought up in large tubs, and ladled deftly and passed round rapidly, all standing while grace is sung, and very heartily it is sung. A few who cannot be induced to eat at table are allowed to sit on the floor; but among 300 there were only five on their haunches, tailor fashion.

The sleeping wards above were arranged in neat military order, the beds covered with a coarse sheet and grey or brown blankets with gay borders. The mattresses are of cocoa fibre, as usual in all institutions here. Solitary cells are rather gloomy and dreary; still, the class of patients must be considered, and, looking at the usual home surroundings of the working class here, they are well fed, lodged, and cared for. Workshops of various kinds employ a large number, also wood-cutting and weeding.

The women's block is very old and dingy-looking, and the refractory women's ward reminds one of an old print of a ward long ago in Bethlehem Hospital; it is a large bare room with a grating running along one side, dividing it from a corridor leading to solitary cells, nurses' pantry, and dormitories. Here about fifty were screaming, singing, moaning, roaming up and down like caged animals, in charge of two attendants.

Beneath this is a large bathroom, very unlike an English bath certainly, but all that is required. If one figures a large room with asphalte floor, several immense tubs of water, and all round the room little tubs for each person, some rough rails with small towels, one has a fair idea of the bathrooms.

The infirmary ward is gay with red covers on the beds, and is bright and cheery. The head warder's quarters at the end overlook all.

On the opposite side of the public road is a small *annexe*, called The Cottage, intended for paying and better-class patients. This is comfortably furnished, and some bright prints on the walls give a more home-like look. A sewing class is held here daily. Most of the female patients are inclined to be noisy, and the males morose.

